# The Role of Working Memory on Dual-Task Cost During Walking Performance in Childhood

**DOI:** 10.3389/fpsyg.2019.01754

**Published:** 2019-07-31

**Authors:** Emanuela Rabaglietti, Aurelia De Lorenzo, Paolo Riccardo Brustio

**Affiliations:** ^1^Department of Psychology, University of Turin, Turin, Italy; ^2^NeuroMuscularFunction Research Group, Department of Medical Sciences, School of Exercise and Sport Sciences, University of Turin, Turin, Italy

**Keywords:** cognitive abilities, motor development, school age, dual-task activity, walking

## Abstract

This study examined the effect of a secondary motor task on walking ability, whether performance differed according to age and the possible relationship between cognitive abilities, specifically working memory, and dual-task costs in children with typical development. Fifty-three female children (mean age *M* = 10 ± 2 years), were divided into two different age groups: a young (7–9 years; *n* = 17) and an older group (10–13 years; *n* = 36). First, participants performed a Walking Test (WT) without additional tasks; afterward, they performed the same walking test while performing each of the following tasks: carrying (1) a glass of water, (2) a ball on a round tray and (3) the combination of both tasks (1) and (2). The Test of Memory and Learning were used to assess working memory. WTs under a dual-task condition generally produced worse results compared to a single-task condition [*F*(3,135) = 32.480, *p* < 0.001]. No age-related difference was observed [*F*(1,45) = 0.497, *p* = 0.485]. Age, digit forward and backward, facial memory, and paired recall accounted altogether for 28.6% of variance in dual-task ability during WT while carrying a glass of water and a ball on a round tray. Specifically, facial memory significantly accounted for the variance of DTC in WTWT (*β* = −0.381, *p* = 0.016). Moreover, a trend toward a statistical significance was observed for digit forward (*β* = −0.275, *p* = 0.085). Results underlined that regardless of the age, a dual-task performance might affect walking performance depending on the required secondary task. Moreover, our results showed the association between working memory skills and dual-task cost in walking ability.

## Introduction

Working memory is an integral component of executive functions (Anderson et al., 2010), responsible for the complex cognitive coordination necessary for everyday life activity (Doherty et al., 2018). In the literature, different multiple definitions were given about working memory depending on the assumed theoretical model (e.g., multiple-component model, time-based resource sharing, and embedded process) (Cowan, 2017; Doherty et al., 2018). One of the most studied frameworks of multi-component system includes the model of Baddeley and Hitch (1974). According to this view, working memory includes four separable but interacting subsystems (Baddeley, 2000, 2012): (1) a supervisory system controlling and regulating the cognitive processes, named central executive, that coordinates separate executive functions such as inhibition, task-switching, and dual-tasking (Doherty et al., 2018). Two slave systems: (2) the phonological loop which includes both verbal inputs subject to a rapid decay and a rehearsal process that can be used to refresh decaying representations (Baddeley, 1996) and (3) the visuospatial sketchpad which processes and maintains visual and spatial material (Baddeley and Lieberman, 1980). Finally, (4) a multidimensional passive storage system, which links information across the two slave systems, the long-term memory storage and the perception, named episodic buffer (Baddeley, 2012). We frame our study within the multi-component working memory model.

Working memory capacity linearly develops across childhood and reaches the adult level during adolescence (Gathercole, 1998; Bathelt et al., 2017), depending on brain structure maturations (Bathelt et al., 2017). The phonological loop appears to be intact in children but, only after age 7, the child increases the amount of verbal material and is able to maintain and rehearse spontaneously (Gathercole et al., 2004). Developmental data for visuospatial sketchpad support the division between visual and spatial components and show an increase in both components as age increases (Swanson, 1999; Mammarella et al., 2008) but with different rates and trajectories (Hamilton et al., 2003).

Working memory conflicts between tasks are a major source of interference in dual tasking (Nijboer et al., 2016). Indeed, dual-task methodology is used to distinguish developmental changes in the different components of working memory (Willis and Gathercole, 2001; Conway et al., 2005; Jaroslawska et al., 2018). Specifically, dual-task methodology requires simultaneous processing and storage demands, such as reading while increasing the series of allowed sentences and eventually recalling the last word of each sentence (Daneman and Carpenter, 1980). In this situation, the simultaneous performances, that rely on the same component of working memory, lead to a competition in cognitive resources with a reduction in working memory capacity (i.e., decrease of the amount of additional information that can be maintained) and less efficient performance when two tasks are separately performed (Daneman and Carpenter, 1980; Jaroslawska et al., 2018). On the contrary, when the performance of two tasks requires the use of two domain-specific slave systems (i.e., use of verbal and visuospatial information) the performance of the two tasks is as efficient as the performance of a single task (Thomason et al., 2009).

Similar to working memory, the development of walking skills occurs according to different processes from the first years of life until adolescence (Shumway-Cook and Woollacott, 2012). In the first years of life, walking is characterized by rapid improvements in gait pattern (Kraan et al., 2017). Mature and independent walking is reached around age 2–3 years. After this, a child shows a refinement of gait pattern, such a reduction in the base of support width, a clear toe-off and heal strikes and reciprocal arm swing (for a review, please consult Kraan et al., 2017), until walking becomes steady and similar to the adult pattern around age 7–8 years (Sutherland, 1997; Kraan et al., 2017). Nevertheless, walking patterns can be refined and improved after this age (Belmonti et al., 2013; Manicolo et al., 2016), also due to the maturation in brain structure (Leisman et al., 2016).

During everyday life activities, single-task motor performances are rarely carried out alone, instead dual-task performances are more common. These situations, including dual-task activities, may lead to a change in one of the two activities and may be particularly challenging during childhood. Indeed, the simultaneous performance of a secondary task, such as a motor or cognitive task or both, together with a primary task, may affect the primary task, such as the walking task (Huang et al., 2003; Ruffieux et al., 2015; Saxena et al., 2017; Brustio et al., 2017b; Schott and Klotzbier, 2018). Attentional resources are limited (Kahneman, 1973; Navon and Gopher, 1979) when shared between the primary and secondary tasks (Wickens, 1991). Consequently, due to a competition of the same pool of cognitive resources (Navon and Gopher, 1979), a worsening of the performance occurs, known as dual-task cost (e.g., there is a reduction of performance in dual-task performance, compared to single-task performance).

Studies on walking tasks underlined a decrease in performance both with additional motor tasks (Huang et al., 2003; Cherng et al., 2007; Hung et al., 2013; Abbruzzese et al., 2014) and cognitive tasks (Schaefer et al., 2015; Schott and Klotzbier, 2018). For example, Cherng et al. (2007) investigated the impact of concurrent motor and cognitive tasks on walking ability in preschool children (age 4–6 years) and showed that the concurrent performance of a secondary task negatively affected the gait parameters, depending on the difficulty and type of the secondary task. However, some studies showed an improvement of dual-task performance with children’s growth, while other studies did not confirm this evidence (for a further review on the topic, see: Saxena et al., 2017). For example, Boonyong et al. (2012) underlined that postural control while performing a walking task under dual-task condition improves as age increases. Differently, Hagmann-von Arx et al. (2016) did not report the effect of age on walking ability with an additional cognitive task. We can assume that the variation in the use of non-standardized test protocols (e.g., different additional tasks) may have affected these results. Despite the above findings, more attentional resources during childhood development are required for walking control (Boonyong et al., 2012; Chauvel et al., 2017).

Walking is one of the most common human activities. However, under dual-task conditions, it is a cognitively demanding activity (Yogev-Seligmann et al., 2008). Different studies investigated the relationship between cognitive resources and walking abilities under dual-task conditions particularly in older people (Yogev-Seligmann et al., 2008), but, to our knowledge, studies on childhood are limited. Using an additional cognitive task during walking performance, Hagmann-von Arx et al. (2016) did not find any association between the ability to walk during dual-task condition and cognitive abilities in childhood. However, there is a gap in the literature regarding the relationship between the cognitive function and walking ability under the dual-task performance with a secondary motor task. Thus, we investigated (1) the effect of a secondary motor task on walking ability, (2) whether performance differed according to age, and (3) the possible relationship between cognitive abilities, specifically working memory, and dual-task costs during children’s typical development. In particular, using the framework of multi-component systems, we wanted to investigate the role, if any, of working memory on walking ability under the dual-task condition. Indeed, given that task-relevant information is controlled, regulated, and actively maintained by working memory (Beurskens and Bock, 2012), it is plausible to speculate that it may be associated with walking ability under the dual-task condition.

## Material and Procedure

### Participants

Fifty-three female children with typical development, aged 7–13 years (mean age *M* = 10 ± 2 years), participated in the study. Children were divided into two different age groups: a young (7–9 years; *n* = 17) and an older group (10–13 years; *n* = 36). All participants were naïve to the purpose of the study; they had normal or corrected-to-normal eye sight, normal hearing and no neuromuscular, neurological, or cardiovascular diseases nor attentional deficits, according to their parents’ reports. The study was conducted in conformity with the recommendations of the Ethics Committee of the University of Torino (Protocol Number: N. 60193). Before starting the study, each parent or legal guardian read, concurred, and signed a written informed consent in accordance with the Declaration of Helsinki.

### Measurements

The Italian version of Test of Memory and Learning (TOMAL; Reynolds and Bigler, 1994; Reynolds et al., 1995) was used to assess cognitive abilities (i.e., memory and learning). The TOMAL is a validated battery designed to assess memory functions (i.e., associative and free recall, verbal memory, attention, and concentration) in children and adolescents, from 5 to 19 years, 11 months and 30 days. TOMAL is composed of 10 core subtests divided into verbal and non-verbal indexes (i.e., five subtests for each index) and four optional supplemental subtests. The combination of these two indexes produces the Composite Memory Index. Internal consistency reliability for each subtest ranges from 0.75 to 0.99 (Cronbach Alpha coefficients) and test-retest reliability ranges from 0.71 to 0.92. For more details about TOMAL subtests see Reynolds and Bigler (1994). The following subtests were used for this study:

1. digit forward: this verbal subtest measures rote learning, sequential memory, and attention and concentration and requires the capability to recall a list of numbers in the correct order. The score was the total number of items correctly repeated. The Cronbach alpha coefficient for ages 7–13 years is 0.97.
2. digit backward: this verbal subtest measures attention, concentration, and the backward span tasks which tap into working memory and requires the capability to recall a list of numbers in reverse order. The score was the total number of items correctly repeated. The Cronbach alpha coefficients for ages 7–13 years of this subtest range from 0.96 to 0.98.
3. facial memory: this subtest measures visual and deferring memory and requires capability to recognize faces from different ethnicities, gender, and age in black and white photos. The score was the total number of recognized photos. The Cronbach alpha coefficients for ages 7–13 years of this subtest range from 0.75 to 0.8.
4. paired recall: this subtest measures learning and associative recall and requires the capability to pair a prompted word with an associated word. The score was the total number of paired words. The Cronbach alpha coefficients for ages 7–13 years of this subtest range from 0.83 to 0.91.

Physical measures included a single-task trial and three different dual-task trials. The single-task trials consisted in the performance of a Walking Test (WT) along a pathway of 14meters. The participants were instructed to walk at their self-selected speed. The walking speed was measured using a digital stopwatch to the nearest 0.01 s. The first and the last meter were not included in the analysis and considered as acceleration and deceleration phases. Each child completed WT while performing. The following additional secondary task (Brustio et al., 2018a,c):

1. carrying a glass of water (filled to 10 mm from the rim) with the preferred hand (WTW);
2. carrying a ball (weight: 100 g, diameter: 7 cm) on a round tray (weight: 50 g, diameter: 17 cm) with the dominant hand only (WTT);
3. carrying a glass of water (filled to 10 mm from the rim) and a ball (weight: 100 g, diameter: 7 cm) on a round tray (weight: 50 g, diameter: 17 cm) with the dominant hand only (WTWT).

Participants first performed WT with a single-task condition followed by WT with a dual-task condition. The WT with a dual-task condition was performed in a counterbalanced order. Dual-task walking conditions included one familiarization trial ahead of the test trial (Hagmann-von Arx et al., 2016). The trial was repeated if any of the above dual-task walking conditions failed (Cherng et al., 2007). No instructions were given regarding which task to prioritize (Brustio et al., 2017a).

Participants were individually tested by the same investigator and evaluated in 1 day, with a 3-min break between each cognitive subtest and walking test.

### Analysis

Repeated measures of analysis of variance (ANOVA) with within-subject factor Task Condition (Task Condition: WT, WTW, WTT, and WTWT) and between-subject factor Age Group (Age Group: young and older group) were run to determine within-subject and age-related differences in walking performance with a dual-task condition. Significant results were followed up by means of *post hoc* analysis with Bonferroni correction.

Dual-task costs (DTC) were calculated as the difference between the scores of the dual-task and single-task performances in order to quantify the participants’ dual-task ability (Brustio et al., 2018b). A positive value of DTC indicates lower dual-task ability while a negative value indicates higher dual-task ability.

Hierarchical multiple regression analyses were conducted to assess the relationship between DTCs and cognitive performance. Specifically, we used each DTC (e.g., DTC in WTW, WTT, and WTWT) as a dependent variable and age, digit forward, digit backward, facial memory, and paired recall as independent factors. Specifically, independent factors were entered in three steps of the regression model with the following order: age in Step 1; age and digit forward and backward subtests (i.e., verbal memory, attention, and concentration) in Step 2; age, digit forward and backward facial memory and paired recall subtests (i.e., associative and free recall) in Step 3.

## Results

**Figure 1:**
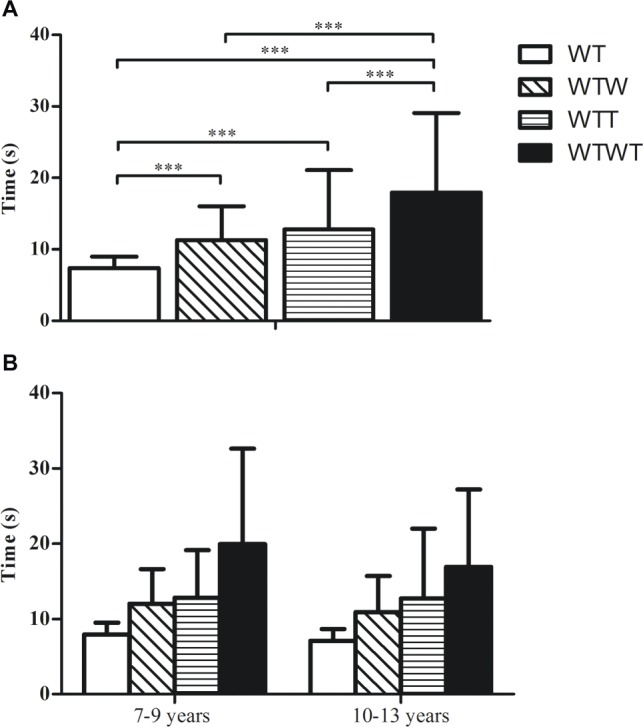
Mean and standard deviation of Walking Test (WT), Walking Test while carrying a glass of water (WTW), Walking Test while carrying a ball on a round tray; Walking Test while carrying a glass of water and a ball on a round tray (WTWT) considering the whole sample **(A)** and the young (7–9 years) and older children group (10–13 years) **(B)**. ^***^p < 0.001.

The test performances are summarized in Table 1 and Figure 1. Overall, a significant difference was observed in Task Condition [*F*(3,135) = 32.480, *p* < 0.001, partial *η*^2^ = 0.419] but not for Age Group [*F*(1,45) = 0.497, *p* = 0.485, partial *η*^2^ = 0.011] and Task Condition × Age Group interaction [*F*(1,45) = 0.256, *p* = 0.615, partial *η*^2^ = 0.006]. *Post hoc* analysis with a Bonferroni adjustment underlined a statistically significant worse walking performance in WTW [3.95 s, 95% CI (2.21, 5.57), *p* < 0.001], WTT [5.28 s, 95% CI (1.98, 8.59), *p* < 0.00], and WTWT [10.57 s, 95% CI (6.44, 15.39), *p* < 0.001] compared to WT. A significant worse walking performance was observed in WTWT compared to WTW [6.96 s, 95% CI (3.58, 10.35), *p* < 0.001] and WTT [5.63 s, 95% CI (3.78, 8.06), *p* < 0.001]. No significant difference was observed between WTW and WTT (*p* > 0.05). For more details, see Figure 1A.

**Table 1:** Results of test cognitive and motor performance.

Variables	*M*	SD
Digit forward (scores)	43.19	14.78
Digit backward (scores)	30.41	17.87
Facial memory (scores)	23.63	5.12
Paired recall (scores)	28.00	5.11
WT (s)	7.38	1.61
WTW (s)	11.29	4.73
WTT (s)	12.79	8.28
WTWT (s)	17.95	11.11
DTC WTW (s)	3.91	1.06
DTC WTT (s)	5.40	7.70
DTC WTWT (s)	10.57	10.48

*M, mean; SD, standard deviation; WT, Walking Test; WTW, Walking Test while carrying a glass of water; WTT, Walking Test while carrying a ball on a round tray; WTWT, WT while carrying a glass of water and a ball on a round tray; DTC, dual-task cost*.

Table 2 presents the results of hierarchical multiple regression analyses obtained in the different steps. Independent factors were not associated with DTC in WTW [*F*(5,44) = 2.201, *p* = 0.074, *R*^2^ = 0.220] nor in WTT [*F*(5,44) = 2.188, *p* = 0.075, *R*^2^ = 0.219]. However, the addition of facial memory and paired recall to the prediction of DTC in WTW (Step 3) led to a statistically significant increase in *R*^2^ of 0.120 [*F*(1, 39) = 3.425, *p* = 0.043]. Differently, independent factors accounted altogether for 28.6% of the variance in DTC in WTWT [*F*(5,44) = 3.130, *p* = 0.018]. The addition of digit forward and backward to the model (Step 2) led to an increase in *R*^2^ of 0.136, *F*(2, 41) = 3.236, *p* = 0.05, while the addition of facial memory and paired recall (Step 3) also led to an increase in *R*^2^ of 0.146, *F*(1, 39) = 3.998, *p* = 0.026. Specifically, facial memory significantly accounted for the variance of DTC in WTWT (*β* = −0.381, *p* = 0.016). Moreover, a trend toward a statistical significance was observed for digit forward (*β* = −0.275, *p* = 0.085).

**Table 2:** Summary of hierarchical regression analyses on dual-task costs.

Dual-task cost	Independent variables	*R*^2^	Δ*R* ^2^	*β*	Partial *r*	*p*
**WTW**						
Step 1	Age	0.001		−0.007	−0.007	0.965
Step 2	Age			−0.043	−0.044	0.778
	Digit forward			−0.317	−0.288	0.061
	Digit backward	0.083	0.082	0.127	0.120	0.442
Step 3	Age			−0.022	−0.023	0.886
	Digit forward			−0.198	−0.192	0.230
	Digit backward			0.279	0.258	0.103
	Facial memory			−0.332	−0.319	0.042
	Paired recall	0.220	0.137	−0.198	−0.188	0.240
**WTT**						
Step 1	Age	0.007		0.081	0.007	0.597
Step 2	Age			0.020	0.044	0.894
	Digit forward			−0.225	0.288	0.174
	Digit backward	0.108	0.101	−0.161	0.020	0.325
Step 3	Age			0.018	−0.114	0.903
	Digit forward			−0.116	−0.016	0.480
	Digit backward			−0.016	−0.239	0.922
	Facial memory			−0.243	−0.235	0.133
	Paired recall	0.219	0.111	−0.250	0.020	0.140
**WTWT**						
Step 1	Age	0.004		−0.066	−0.066	0.665
Step 2	Age			−0.126	−0.132	0.399
	Digit forward			−0.392	−0.358	0.018
	Digit backward	0.140	0.136	0.055	0.054	0.729
Step 3	Age			−0.086	−0.095	0.553
	Digit forward			−0.275	−0.272	0.085
	Digit backward			0.199	0.196	0.219
	Facial memory			−0.381	−0.375	0.016
	Paired recall	0.286	0.146	−0.134	−0.134	0.402

*WTW, walking test while carrying a glass of water; WTT, walking test while carrying a ball on a round tray; WTWT, walking test while carrying a glass of water and a ball on a round tray*.

## Discussion

The aims of this study were to investigate (1) the effect of a secondary motor task on walking performance, (2) whether children’s performance differed according to age, and (3) the possible relationship between cognitive abilities, specifically working memory, and dual-task costs in children with typical development. For this purpose, we evaluated the concurrent performance of walking with different secondary motor tasks, i.e., while carrying a glass of water (WTW), while carrying a ball on a round tray (WTT), and while carrying a glass of water and a ball on a round tray, using the preferred hand (WTWT). Results underlined that an additional task may affect walking performance depending on the required secondary task. Moreover, no age-related differences in dual-task performance were observed. Finally, our results showed the association between facial memory skills and walking ability in dual-task performance while carrying a glass of water and a ball on a round tray.

### Walking Performance During Dual-Task

Considering the primary aim of the study, our results found that dual-task walking performances were generally worse when compared to a single-task condition (see Figure 1), showing a significant effect on the additional motor task. The observed changes may be attributed to the limited cognitive resources in children’s information-processing capability (Granacher et al., 2011; Hung et al., 2013). Because of the development process in postural control ability, the walking and dynamic balance performances are impaired (Granacher et al., 2011). Consequently, children require an increase in cognitive resources to maintain the postural control under dual-task conditions (Ruffieux et al., 2015). Interestingly, WTWT was higher compared to WTW and WTT, suggesting that walking performance under a dual-task depends on the level of difficulty (Patel et al., 2014; Brustio et al., 2017b; Schott and Klotzbier, 2018).

### Age-Related Difference in Walking Performance During Dual-Task

Our results suggested a similar trend in dual-task performance among young and older groups. In other words, we found that, independently from the additional required motor task, both young and older children manage their walking performance similarly. Using a walking task with additional motor tasks (i.e., carrying a box) Hung et al. (2013) showed that younger children (4–6 years old) had less bimanual coordination, as well as greater difficulty in walking task, compared to 7–13 years old children. On the contrary, no difference was observed between a younger (7–9 years old) and an older group (10–13 years old) under dual-task constraints (Hung et al., 2013). Indeed, a weak evidence of interference in younger children (2–6 years) compared to older children (7 and older) appeared when the task conditions were difficult or complex (Saxena et al., 2017).

### Relationship Between Working Memory and Dual-Task Costs

When focusing on the relationship between cognitive abilities and dual-task costs, we found a negative relation between DTC (i.e., carrying a glass of water) and visual memory and recall (i.e., facial memory subtests). It is possible to speculate that during WTW children used the non-verbal information of the visuospatial sketchpad processed by the central executive (e.g., visual information storage and processing) to manage two independent streams of visual information, one related to the walking task and the other related to the secondary task (Beurskens and Bock, 2012).

Differently from the WTW costs, when the difficulty of the secondary task increased (i.e., carrying a glass of water and a ball on a round tray) we found a negative association with visual memory and recall and a negative trend with verbal memory, rote learning, attention, and concentration (i.e., digit-forward subtest). Thus, in order to manage the two concurrent tasks, both the visuospatial sketchpad processing by the central executive and the elaboration of verbal information were involved. Indeed, according to Luria (1961) and Vygotsky (1934) when children perform complex tasks (i.e., multitasking), they activate the regulatory role of language, i.e., inner speech (Emerson and Miyake, 2003), to support more demanding executive tasks (Baddeley, 2012). Inner speech plays a role in task switching performance and is associated with the phonological loop system and supports the executive control process in Baddeley’s working memory model (Emerson and Miyake, 2003). Contrary to our results, Hagmann-von Arx et al. (2016) found no relationship between spatial working memory and walking ability during dual-task performance. Their results may have been influenced by the different nature of the secondary task: either listening to and recalling digits or pressing a button as well as by the use of differently used cognitive tests.

### Limitations

Our study presents some limitations. Due to the relatively small sample size of the study, we were not able to extend our conclusions to the larger children’s population and to study age-related differences. Moreover, the cross-sectional nature of the study did not allow us to investigate the possible causal relationship between cognitive and physical ability. Moreover, we evaluated walking performance by means of a single parameter (time), and the secondary tasks used in the present study were motor tasks only. Another limitation to our study was that single and dual-task conditions were not counterbalanced. Thus, our participants first performed the walking performance as a single-task, followed by dual-task conditions. Therefore, potential practice or fatigue effects were not controlled. As previously suggested (Schott and Klotzbier, 2018), it is possible that counterbalancing may have increased the magnitude of the observed differences among single and dual-task performances. Finally, our primary goal was to investigate the effect of a secondary task on walking performance and therefore the secondary task was not evaluated. Thus, no data on bidirectional dual-task cost were provided.

## Conclusion

In conclusion, our results show that regardless of age, a dual-task performance may affect walking ability, depending on the required secondary task. Moreover, our results showed an association between working memory skills and dual-task cost in children with typical development.

## Data Availability

The datasets generated for this study are available on request to the corresponding author.

## Ethics Statement

The study was conducted in conformity with the recommendations of the University of Torino Ethics Committee. Before starting the study, each parent or legal guardian read, concurred and signed a written informed consent in accordance with the Declaration of Helsinki. The Ethical Committee of the University of Torino approved the study (Protocol Number: N. 60193).

## Author Contributions

ER, AD, and PB contributed to conceptualization. AD and PB contributed to investigation. PB contributed to formal analysis. ER, AD, and PB contributed to writing – original draft. ER, AD, and PB contributed to writing – review and editing. ER contributed to supervision.

### Conflict of Interest Statement

The authors declare that the research was conducted in the absence of any commercial or financial relationships that could be construed as a potential conflict of interest.
